# Nanomedicine-driven microRNA therapy: a precision approach for eosinophilic granulomatosis with polyangiitis

**DOI:** 10.1097/MS9.0000000000004036

**Published:** 2025-10-07

**Authors:** Ghanwa Imran, Anusha Kumari, Maliha Khalid, Muhammad Talha, Aminath Waafira

**Affiliations:** a Department of Medicine, Lahore Medical and Dental College, Lahore, Pakistan; b Department of Medicine, Jinnah Sindh Medical University, Karachi, Pakistan; c Department of Medicine, King Edward Medical University, Lahore, Pakistan; d School of Medicine, The Maldives National University, Malé, Maldives

**Keywords:** eosinophilic granulomatosis with polyangiitis, microRNA therapy, nanomedicine, precision medicine

## Abstract

Eosinophilic granulomatosis with polyangiitis (EGPA), the rarest form of ANCA-associated vasculitis, remains a challenging condition to manage due to its complex pathophysiology involving eosinophilia, granulomatous inflammation, and small- to medium-vessel necrotizing vasculitis. Emerging evidence suggests that microRNAs (miRNAs), which show distinct patterns of upregulation and downregulation in EGPA patients, play a critical role in disease activity and progression. Advances in nanomedicine have enabled targeted delivery of RNA-based therapies, including antisense oligonucleotides, small interfering RNAs, and therapeutic miRNAs, directly to immune and endothelial cells. Recent findings, such as the identification of LINC02193 as a microRNA sponge correlated with disease activity, highlight their diagnostic and therapeutic promise. While miRNA-based nanotherapies represent a precision approach capable of modulating disease-specific pathways with fewer side effects, their clinical translation requires further research to validate safety, efficacy, and long-term outcomes.

*To the Editor,*

Eosinophilic granulomatosis with polyangiitis, also known as Churg–Strauss syndrome, is a rare disorder and is now recognized as one of the anti-neutrophil cytoplasmic antibody (ANCA) associated vasculitides (AAV). EGPA is the rarest form of vasculitis, with a prevalence of 0.9 to 2.4 per million. It is characterized by necrotizing granulomatous inflammation of small to medium-sized vessels with few or no immune deposits, along with asthma and eosinophilia of more than 10%. Other criteria for its diagnosis include mono or poly neuropathy, non-fixed pulmonary infiltrates, paranasal sinus abnormality, and extravascular eosinophilia. Median age of its occurrence is 49–59 years^[1]^.

In patients with vasculitis, microRNAs are differently expressed in exosomes compared to healthy individuals, which could be a target point for therapy in these patients. These exosomal mRNAs show upregulation of transcripts such as DEPDC1B and TPST1, and downregulation of transcripts like NSUN4 and AK4. This represents the underlying molecular change, which is related to the pathophysiology of this disease. By targeting these altered microRNAs, either through RNA interference methods such as antisense oligonucleotides or small interfering RNAs (siRNAs) or through therapeutic microRNAs (miRNAs) that control their expression, a promising therapeutic approach is guaranteed. Nanomedicine delivery technique has now made it possible to deliver targeted RNA therapies to specific immune and endothelial cells involved in vasculitis^[2]^.

Recent clinical studies emphasized the importance of using microRNAs as a tool to differentiate between patients with vasculitis and healthy controls, indicating their therapeutic potential. A recent mechanistic analysis has highlighted the discovery of a new microRNA sponge, LINC02193, and found it to be significantly correlated with disease activity, according to the Birmingham Vasculitis Activity Score (BVAS), as well as with the neutrophil cytoplasmic ratio. It also correlates with ESR and CRP levels, making it a valuable target for RNA therapy^[3]^. Another recent study discovered that certain microRNAs are upregulated and downregulated in the plasma of patients with the disease compared to healthy controls, demonstrating their clinical importance^[4]^.

Although most recent studies have identified four most important microRNAs that can serve as potential therapeutic targets, further studies are required to establish the exact correlation between these microRNAs and the pathogenesis of the disease. Also, their diagnostic and therapeutic value in disease progression remains unknown, and their patient safety profile has yet to be determined. Given the complexity of vasculitic disorders, including Churg–Strauss syndrome, further studies are required to understand the role of target microRNAs in disease advancement. Lastly, the implications of all these findings in a practical clinical scenario are yet to be determined^[5]^.

In conclusion, microRNAs hold significant potential as therapeutic agents, offering a promising avenue for the treatment of a wide range of chronic diseases, including vasculitides. Their ability to regulate gene expression at the molecular level can also be utilized to halt the progression of the disease. miRNAs form the basis of precision therapy by targeting specific molecules and pathways, thereby minimizing side effects. However, further research is essential to ensure their safe and effective application in clinical practice. This letter to editor is in compliance with the TITAN guideline^[6]^.

## Data Availability

None.
